# A review of chemotherapeutic drugs-induced arrhythmia and potential intervention with traditional Chinese medicines

**DOI:** 10.3389/fphar.2024.1340855

**Published:** 2024-03-20

**Authors:** Weina Li, Xiaozhen Cheng, Guanghui Zhu, Ying Hu, Yunhan Wang, Yueyue Niu, Hongping Li, Aikeremu Aierken, Jie Li, Ling Feng, Guifang Liu

**Affiliations:** ^1^ Guang’anmen Hospital, China Academy of Chinese Medical Sciences, Beijing, China; ^2^ First Teaching Hospital of Tianjin University of Traditional Chinese Medicine (National Clinical Research Center for Chinese Medicine Acupuncture and Moxibustion), Tianjin, China; ^3^ Henan Province Hospital of Traditional Chinese Medicine (The Second Affiliated Hospital of Henan University of Traditional Chinese Medicine), Zhengzhou, Henan, China

**Keywords:** chemotherapeutic drugs, traditional Chinese medicine, arrhythmia, adverse effects, review

## Abstract

Significant advances in chemotherapy drugs have reduced mortality in patients with malignant tumors. However, chemotherapy-related cardiotoxicity increases the morbidity and mortality of patients, and has become the second leading cause of death after tumor recurrence, which has received more and more attention in recent years. Arrhythmia is one of the common types of chemotherapy-induced cardiotoxicity, and has become a new risk related to chemotherapy treatment, which seriously affects the therapeutic outcome in patients. Traditional Chinese medicine has experienced thousands of years of clinical practice in China, and has accumulated a wealth of medical theories and treatment formulas, which has unique advantages in the prevention and treatment of malignant diseases. Traditional Chinese medicine may reduce the arrhythmic toxicity caused by chemotherapy without affecting the anti-cancer effect. This paper mainly discussed the types and pathogenesis of secondary chemotherapeutic drug-induced arrhythmia (CDIA), and summarized the studies on Chinese medicine compounds, Chinese medicine Combination Formula and Chinese medicine injection that may be beneficial in intervention with secondary CDIA including atrial fibrillation, ventricular arrhythmia and sinus bradycardia, in order to provide reference for clinical prevention and treatment of chemotherapy-induced arrhythmias.

## 1 Introduction

Cancer is currently the leading cause of human disease-related death worldwide, and the incidence and mortality of cancer are increasing year by year (Sung et al., 2021). Major advances in chemotherapy drugs have reduced the mortality of malignant tumors, however, chemotherapy-related cardiac toxicity has increased the morbidity and mortality of patients, and has become the second leading cause of death after tumor recurrence, which has received more and more attention in recent years (Curigliano et al., 2016; Zamorano et al., 2016). Arrhythmia is one of the common types of chemotherapy-induced cardiotoxicity and has become a new risk associated with chemotherapy (Alexandre et al., 2021; Garg and Fradley, 2021), seriously affecting patient outcomes (Tamargo et al., 2015). In addition, the incidence of cancer is closely related to increasing age, so the prevention and treatment of chemotherapeutic drug-induced arrhythmia (CDIA) appear to be very important, especially in improving the quality of life of elderly patients. CDIA can be classified into the primary (Drugs directly disrupt specific molecular targets or signaling pathways that regulate the electrophysiological properties of the heart muscle) and the secondary (caused by ischemia, inflammation, oxidative stress, ER stress, apoptosis damage to the endocardium/myocardium/pericardium, which is a secondary phenomenon) (Buza et al., 2017). Common drugs that cause primary CDIA are Anthracyclines (Buza et al., 2017), Immunomodulatoryb Drugs (Rajkumar et al., 2003), Multitargeted Tyrosine Kinase Inhibitors (Buza et al., 2017). Common drugs that cause secondary CDIA are Anthracyclines (Buza et al., 2017), Antimetabolites (Khan et al., 2012), Platinum Compounds (Thix et al., 2009), Proteasome Inhibitors (Buza et al., 2017), Multitargeted Tyrosine Kinase Inhibitors (Buza et al., 2017), Vascular Endothelial Growth Factor Signaling Pathway Inhibitors (Buza et al., 2017), Human Epidermal Growth Factor Receptor 2/neu Inhibitors (Piotrowski et al., 2012), Immune Check Point Inhibitors (Heinzerling et al., 2016; Behling et al., 2017). The secondary CDIA is much more common in the clinic (see Table 1 for CDIA classification and underlying mechanisms).

**TABLE 1:** Primary and secondary arrhythmias due to chemotherapy-related cardiotoxicity.

Classification of CDIA	Classification of chemotherapeutic agents	Cardiotoxicity endpoint	References
Primary	Anthracyclines	QTc interval prolongation	Buza et al. (2017)
	Thalidomide	——	Rajkumar et al. (2003)
	Alectinib, Ceritinib, Crizotinib, Ibrutinib, Ponatinib	QTc interval prolongation	Buza et al. (2017)
	Cisplatin	QTc interval prolongation	Thix et al. (2009)
Secondary	Anthracyclines	Cardiomyopathy	Buza et al. (2017)
	5-Fluorouracil	Myocardial ischemia	Khan, et al. (2012)
	bortezomib, carfilzomib	Heart failure	Buza et al. (2017)
	Dasatinib	Cardiomyopathy	Buza et al. (2017)
	sunitinib	Heart failure	Buza et al. (2017)
	Trastuzumab	Left ventricular systolic dysfunction	Piotrowski et al. (2012)
	Pembrolizumab, nivolumab	Myocarditis	Heinzerling et al. (2016), Behling et al. (2017)

——: Not mentioned.

Traditional Chinese medicine has experienced thousands of years of clinical practice in China, and has accumulated a wealth of medical theories and treatment formulas, which has unique advantages in the prevention and treatment of malignant diseases, such as arsenic trioxide in the treatment of acute promyelocytic leukemia (Chen et al., 2021; Kayser et al., 2021). Traditional Chinese medicine may reduce the arrhythmic toxicity caused by chemotherapy without affecting the anti-cancer effect (Yang et al., 2018). This paper mainly discussed the types and pathogenesis of secondary CDIA, and summarized the preclinical studies of Chinese medicine compounds, Chinese medicine Combination Formula and Chinese medicine injection on the cause of chemotherapeutic drugs-induced cardiotoxicity, in order to provide reference for clinical prevention and treatment of CDIA.

## 2 Types of arrhythmias caused by chemotherapy drugs

The potential cardiotoxicity of chemotherapy drugs can lead to a variety of arrhythmias (tachyarrhythmic or bradyarrhythmic). The actual incidence of CDIA may be far underestimated because routine cardiac monitoring are not performed or only 12-lead electrocardiograms during clinical therapy (Herrmann, 2020). It is crucial for cardiologists and oncologists to recognize the causal association between chemotherapeutic agents and arrhythmias and to target prevention and treatment for cancer patients.

### 2.1 Atrial fibrillation

Atrial fibrillation (AF) is a common cardiotoxicity of anticancer therapy in cancer patients (Tamargo et al., 2015), especially in the elderly on combination drugs, in patients with advanced cancer, and in patients undergoing active treatment (O'Neal et al., 2015), where the development of AF is a poor prognostic factor (Tamargo et al., 2015). The tyrosine kinase inhibitor (TKI) ibrutinib has the strongest correlation with atrial fibrillation (paroxysmal and persistent), which can occur in 3%–16% (mean 8%) of patients with cancer (Leong et al., 2016; Thompson et al., 2016; Yun et al., 2017; Ye et al., 2021), with incidence rates ranging from 10% to 16% with longer follow-up (Burger et al., 2015; Byrd et al., 2015; Wang et al., 2015; Leong et al., 2016; Brown et al., 2017), and leads to early discontinuation of therapy in 46% of patients with cancer (Byrd et al., 2015). Anthracyclines at standard doses can induce both acute and delayed AF, with the type of AF being predominantly persistent (Ando et al., 2000; Kilickap et al., 2007; Guglin et al., 2009; Amioka et al., 2016). Cisplatin, when administered intravenously, intrapericardial, or intrathoracically, can cause acute AF in 10%–32% of patients (Tomkowski and Filipecki, 1997; Tomkowski et al., 2004; Bischiniotis et al., 2005; Lara et al., 2005; Richards et al., 2006; Tilleman et al., 2009; Zellos et al., 2009). A systematic review showed that standard doses of gemcitabine, immune checkpoint inhibitors, Marfan, abiraterone acetate, and rituximab can increase the incidence of atrial fibrillation in elderly patients, those with reduced renal function, or those with hypertension (Olivieri et al., 1998; Phillips et al., 2004; Mileshkin et al., 2005; Feliz et al., 2011; Passalia et al., 2013; Sharma et al., 2017; Hilmi et al., 2020; Mascolo et al., 2023). In addition, high doses of cyclophosphamide and isocyclophosphamide increase the risk of paroxysmal AF (Kupari et al., 1990; Quezado et al., 1993).

### 2.2 Ventricular arrhythmias

Arsenic trioxide is considered to be the most dangerous drug causing QTc interval prolongation, as tip-twisting ventricular tachycardia is a very problematic malignant arrhythmic event (Roboz et al., 2014; Zamorano et al., 2016). Immune checkpoint inhibitors can cause ventricular arrhythmias in 5%–10% of patients and lead to a 40% mortality rate (Drugs FDA: 2023 FDA Approved Drug Products. Retrieved September 25). Paclitaxel and ibrutinib can lead to polymorphic ventricular tachycardia, ventricular fibrillation, and even sudden death (Arbuck et al., 1993; Wallace et al., 2016), in a significant dose-dependent manner (Brouty-Boye et al., 1995). 5-Fluorouracil can lead to a significant increase in premature ventricular contraction and ventricular tachycardia events in patients (Yilmaz et al., 2007; Khan et al., 2012; Polk et al., 2013). Adriamycin in combination with 5-fluorouracil can lead to an increase in the incidence of premature ventricular contractions from 3% to 24% (Steinberg et al., 1987). Anthracyclines can lead to acute ventricular arrhythmic events in patients (Tamargo et al., 2015).

### 2.3 Sinus bradycardia

Sinus bradycardia is an important risk factor for prolonged QT interval and the development of lethal polymorphic ventricular tachycardia (Roden, 2016; Zamorano et al., 2016). It is often underestimated clinically, because of its low incidence and the fact that most patients are asymptomatic; therefore, the focus should be on the potentially harmful effects of sinus bradycardia in cancer patients (Font et al., 2023). Thalidomide causes sinus bradycardia (heart rate of 30–50 beats/min) in 53% of patients with multiple myeloma, heart rate as low as 30 beats/min in eight patients with symptoms of severe weakness and hypotension, and syncope in 12% of patients, one-third of whom were fitted with pacemakers (Fahdi et al., 2004; Barlogie et al., 2006; Herrmann, 2020). Sinus cardiac arrest has been reported with ibrutinib (Mathur et al., 2017). A systematic review demonstrated that immune checkpoint inhibitors can cause atrioventricular block or conduction disease in 10% of patients, leading to a 50% mortality rate (Mir et al., 2018). The mean maximum heart rate in patients with non-small cell lung cancer with crizotinib decreased to 25 beats/min, with 18.8% of patients having a heart rate of 45–49 beats/min (Ou et al., 2016).

## 3 Molecular mechanisms of chemotherapy-related cardiotoxicity

**FIGURE 1:**
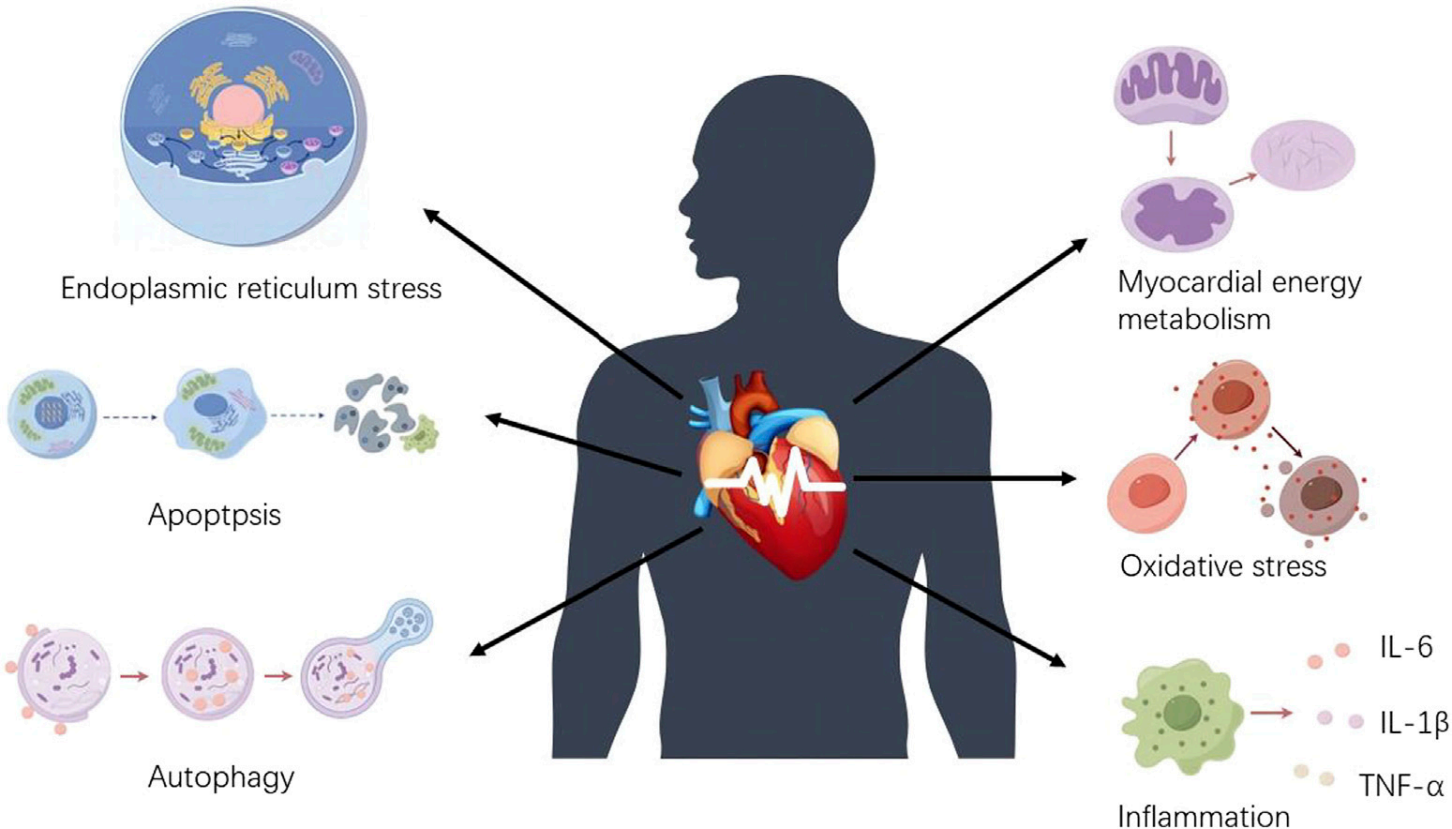
Mechanisms of chemotherapeutic drug-induced arrhythmias. Chemotherapeutic drug-induced arrhythmias are mainly caused by increased oxidative stress, abnormal myocardial energy metabolism, endoplasmic reticulum stress disorder, apoptosis, inflammatory stimuli, and autophagy dysfunction.

**FIGURE 2:**
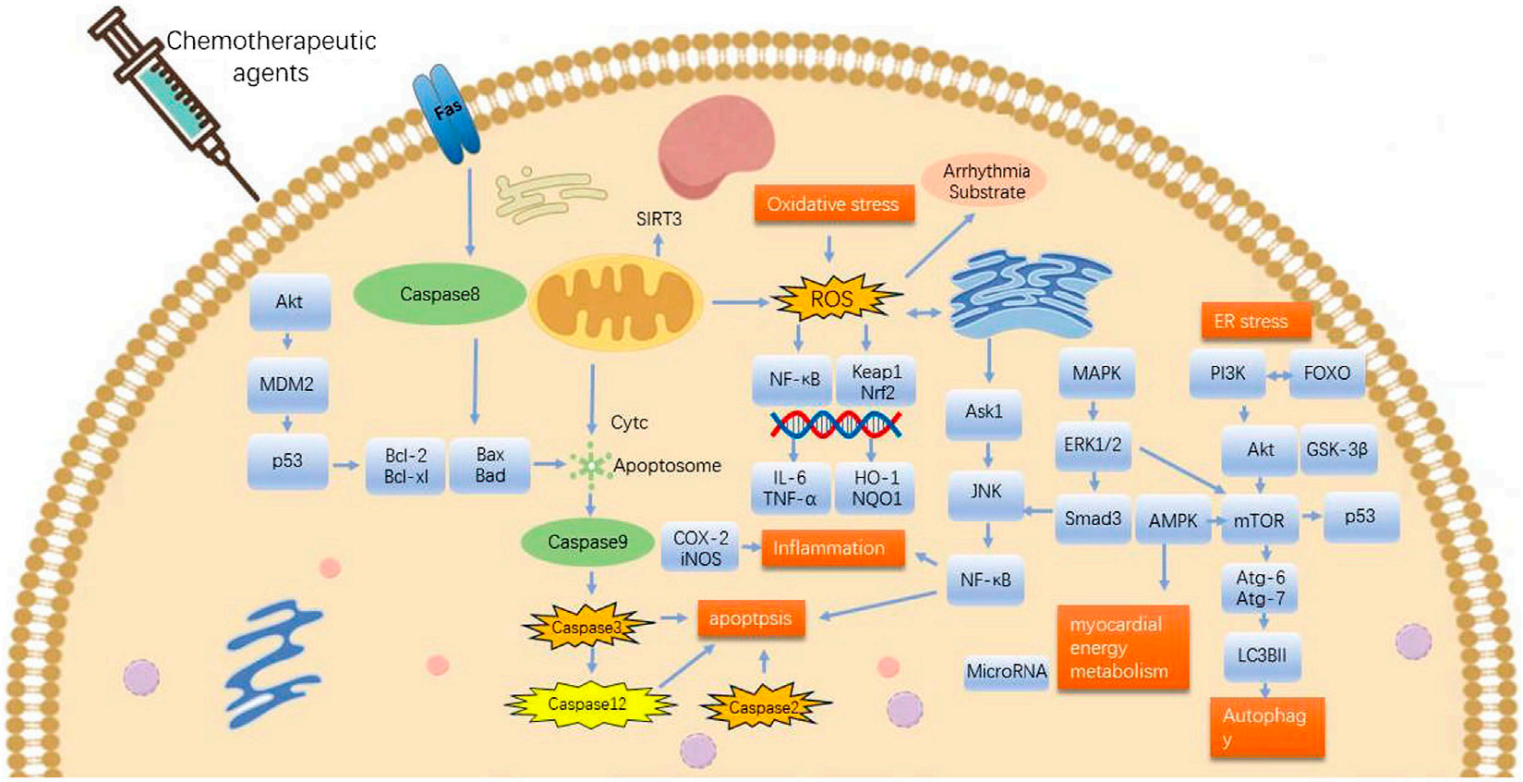
Mechanisms involved in potential effects of TCMs on arrhythmia originating from chemotherapeutic drugs-induced cardiac cytotoxicity. ROS, reactive oxygen species; MAPK, mitogen-activated protein kinase; Nrf2, nuclear factor E2-related factor-2; HO-1, heme oxygenase-1; Keap1, E3 ligase junction Kelch-like ECH-associated protein 1; ER stress, endoplasmic reticulum stress; JNK,c-Jun N-terminal kinase. Pro-survival: Bcl-2, HO-1, AMPK, PI3K, Akt, mTOR, p-AMPK, SIRT3, microRNA, GSK-3β, Nrf2, NQO1, ERK1/2. Pro-death: Keap1, Bid, GSK-3β, caspase-8, caspase-9, ROS, COX-2, iNOS, NF-κB, TNF-α, IL-6, Bax, Caspase-3, ERK1/2, p53, MAPK, FOXO, p38-MAPK, TGFβ1, αSMA, FFA, caspase-12, caspase-2, ERS, JNK-mTOR.

The mechanism of arrhythmia induced by chemotherapeutic agents is complex. This article focuses on CDIA secondary to myocardial injury or cardiac cytotoxicity. Indeed, the secondary CDIA is the result of multifactorial interactions, mainly targeting cell survival and death pathways, and is associated with multiple molecular and signaling pathways, with oxidative stress and inflammation being the most common mechanisms, followed by apoptosis, autophagy, endoplasmic reticulum stress and abnormalities in myocardial energy metabolism, which together contribute to the onset and development of arrhythmias (Figures 1, 2).

### 3.1 Oxidative stress

Oxidative stress (OS) describes a state of imbalance between antioxidants and oxidants in the body, which is triggered when large amounts of Reactive oxygen species (ROS) and their metabolites accumulate intracellularly. Cardiomyocytes are sensitive to chemotherapeutic drug-induced oxidative stress injury because of the abundance of mitochondria, which not only generate ROS but are also the target of ROS-activated injury. OS can cause arrhythmias by altering ion channels, leading to mitochondrial dysfunction and abnormal energy metabolism in cardiomyocytes, inducing cardiac electrical remodeling. Therefore, OS is considered to be the main mechanism causing cytotoxicity. ROS along with OS is a key contributor to arrhythmias and it plays a crucial role in the progression of AF (Barangi et al., 2018; Mason et al., 2020; Yang et al., 2020). It also causes life-threatening arrhythmias such as ventricular tachycardia and ventricular fibrillation (Szyller et al., 2022) and is considered to be the primary mechanism causing cytotoxicity (Sies, 2015; Sinha and Dabla, 2015; Tocchetti et al., 2019; Zhao et al., 2023). Chemotherapy leads to ROS production through multiple pathways, and overproduction of ROS is considered the most prevalent theoretical pathway by which chemotherapy triggers cardiotoxicity (Rochette et al., 2015; Curigliano et al., 2016; Yang et al., 2018; Yang et al., 2020). Available studies suggest a direct or indirect relationship between OS and anticancer drug-induced cardiotoxicity (Zhang et al., 2019; Attanasio et al., 2021). Literature has reported that 5-fluorouracil (5-FU) (Focaccetti et al., 2015) can lead to elevated intracellular OS in cardiomyocytes, thereby causing damage to cardiomyocytes (Durak et al., 2000; Lamberti et al., 2014; Polk et al., 2014; Sara et al., 2018; Rapa et al., 2021). Elevated OS is also an important factor in doxorubicin (Dox)-induced cardiotoxicity (Harstad and Klaassen, 2002; Ghibu et al., 2012; Hussein, 2012; Vejpongsa and Yeh, 2014; Akolkar et al., 2017). Dox promotes the production of ROS in cells and induces cell poisoning (Carresi et al., 2018; Wei et al., 2018), while high levels of ROS promote autophagy and apoptosis by activating p53 (Saha et al., 2014; Shi et al., 2014) and MAPK (Beccafico et al., 2015; Liu et al., 2015) signaling pathways. Nuclear factor E2-related factor-2 (Nrf2), an important transcription factor regulating OS (Zhao et al., 2023), coordinates antioxidant and cytoprotective effects (Wang et al., 2015; Zhao et al., 2023), and is protective against Dox-induced myocardial OS (Yarmohammadi et al., 2021). Nrf2 can enhance endogenous antioxidant protection mechanisms during OS development and through the activation of heme oxygenase-1 (HO-1) for OS protection (Edwardson et al., 2015). Deletion or inhibited expression of Nrf2 leads to abnormal cardiomyocyte remodeling and increased Oxidative Stress (Singh et al., 2015). The Keap1-Nrf2 pathway formed by Nrf2 and its major negative regulator, the E3 ligase junction Kelch-like ECH-associated protein 1 (Keap1), is an important endogenous antioxidant signaling pathway important in attenuating myocardial injury (Li et al., 2014; Wang et al., 2015; Lu et al., 2016; Cuadrado et al., 2018), which may be a therapeutic target for attenuating Dox-induced cardiotoxicity through activation of cellular-level antioxidant defense mechanisms in cardiomyocytes (Arunachalam et al., 2021).

### 3.2 Myocardial energy metabolism

Myocardial energy metabolism is a key factor affecting cardiac function (Doenst et al., 2013; Lopaschuk et al., 2021). Highly active cardiomyocytes must have sufficient energy metabolism to maintain normal function (Yu et al., 2023). Mitochondria are the center of energy metabolism in the organism, and in recent years, it has been found that decreased mitochondrial function caused by abnormal regulation of mitochondrial mass may play an important role in cardiac damage caused by disorders of energy metabolism (Li et al., 2023). AMPK, as the main energy receptor of the cell, plays a central role in this process, just like a “fuel gauge" (Hardie and Carling, 1997), is recognized as an important kinase in the regulation of myocardial energy metabolism, which shows metabolic changes in heart failure (Kitani et al., 2019). Activation of AMPK promotes energy metabolism, increases antioxidant enzyme activity, decreases OS, protects cardiomyocytes from apoptosis, promotes autophagy, and attenuates inflammatory responses (Cai et al., 2022; Zhang et al., 2022; Pasini et al., 2023). Cardiotoxic chemotherapeutic agents have been shown to impair the intracellular mechanisms controlling cardiac metabolism (Fazzini et al., 2022). AMPK has now been found to lie at the control point for many of the mechanisms that have been shown to be associated with Dox cardiotoxicity (Timm and Tyler, 2020). Dox markedly inhibits AMPK activity in the myocardium and can exacerbate myocardial toxicity (Timm and Tyler, 2020). Previous studies have found that trastuzumab causes mitochondrial dysfunction in the human induced pluripotent stem cell model (iPSC-CM) and affects the cardiac energy metabolism pathway (Necela et al., 2017; Kitani et al., 2019), which is consistent with the role of the AMPK pathway in Dox-induced myocardial toxicity (Sharma et al., 2018).

### 3.3 Endoplasmic reticulum stress

The endoplasmic reticulum (ER) is a structurally and functionally diverse membrane-bound organelle in the cytoplasm of eukaryotic cells, which is mainly involved in protein synthesis, folding, maturation, modification, transport and calcium homeostasis (Omidkhoda et al., 2019; Shakeri et al., 2019; Yang et al., 2020; Yarmohammadi et al., 2021). Endoplasmic reticulum stress (ERS) is a “double-edged sword,” which is an adaptive response to maintain cell survival by regulating endoplasmic reticulum function. Prolonged severe or persistent endoplasmic reticulum stress and protein misfolding can induce inflammatory responses and apoptosis, and promote myocardial remodeling (Oakes and Papa, 2015; Narezkina et al., 2021; Ren et al., 2021; Ren et al., 2022). Endoplasmic reticulum stress (ERS) plays a key role in a variety of cardiovascular diseases, including fatal arrhythmias (Sirish et al., 2021; Hamilton and Terentyev, 2022). Recent studies have documented that ERS plays a crucial role in the pathophysiologic basis of AF (Sirish et al., 2021). It has been shown that the ERS pathway regulates a variety of myocardial functions such as inflammatory response, apoptosis, and autophagy. Chronic ERS interferes with the redox state in the endoplasmic reticulum and reduces the expression of myocardial ion channel proteins, which leads to arrhythmogenesis (Hamilton and Terentyev, 2022). The importance of ER and its signaling pathway in Dox-induced cardiotoxicity concerning inflammation, apoptosis, and autophagy suggests that it may be a key factor in the cardiotoxic effects of Dox-induced cardiovascular toxicity (Minamino et al., 2010; Abdel-Daim et al., 2017; Akolkar et al., 2017; Wang et al., 2018; Yarmohammadi et al., 2021). Thus, control of ER and its signaling pathways can significantly slow the development of Dox-induced cardiotoxicity (Yarmohammadi et al., 2021).

### 3.4 Apoptosis

Apoptosis is a highly regulated mode of programmed cell death (Shabalala et al., 2017), in which nucleic acid endonucleases and cysteine-aspartate proteases (caspases) are the most critical enzymes mediating apoptosis, and either insufficient or excessive apoptosis can lead to a variety of diseases. Apoptosis plays an important role in cardiovascular diseases (Michalson et al., 2018; Yang et al., 2020). It has been shown that in cardiomyocytes and endothelial cells, elevated reactive oxygen species triggers caspase-3 and activates apoptosis (Eskandari et al., 2015; Focaccetti et al., 2015; Rawat et al., 2021). Cisplatin opens mitochondrial permeability channels, allowing intra-mitochondrial Cyt-c to enter the cytosol, initiating a mitochondria-dependent pathway and inducing apoptosis (Kim et al., 2003). p53 is an important regulator of cell death and has long been known to be associated with Dox-induced myocardial toxicity (Christidi and Brunham, 2021). Dox can activate the p53 pathway to initiate endogenous, exogenous, and endoplasmic reticulum-associated apoptotic pathways, thereby inhibiting cardiomyocyte apoptosis (Yarmohammadi et al., 2021). In transcriptomic studies, p53 has been shown to be a key transcriptomic regulator of cardiotoxicity (McSweeney et al., 2019). Increased expression of p53-mediated cell death receptors regulates cardiomyocyte apoptosis, autophagy, and leads to cardiac atrophy. It has been reported in the literature that Dox activates p53 in mitochondria, which in turn induces cardiomyocyte apoptosis. It has been found that p53 is overexpressed in cardiomyocytes and can promote apoptosis through transcriptional activation into the nucleus (Wang et al., 2013). Conversely, inhibition of p53 activity promotes apoptosis in cardiomyocytes at the later stages of Dox treatment (Guo et al., 2023). The expression and regulation of Bcl-2 family genes have a decisive influence on the occurrence of apoptosis, among which Bax and Bcl-2 have an important role in the regulation of mitochondrial apoptosis (Qi et al., 2022). These proteins may regulate apoptosis through the PI3K/Akt pathway and the P53 pathway (Wang et al., 2015; Wenningmann et al., 2019). Dox upregulates the expression of Bax and Caspase-9 and downregulates the expression of the Bcl-2 gene, which induces apoptosis in rat myocardial H9c2 cells (Han et al., 2008; Mei et al., 2015; Qi et al., 2022). It has been shown that the mitogen-activated protein kinase (MAPK) signaling pathway plays an important role in cell proliferation, differentiation, and apoptosis (Kim and Choi, 2015; Zhao et al., 2022), c-Jun N-terminal kinase (JNK) belongs to the MAPK family, and Dox induces apoptosis through the JNK and MAPK signaling pathways (Das et al., 2011). It is known that nuclear factor-κB (NF-κB) activation and MAPK signaling pathway can activate pro-apoptotic events leading to apoptosis (Sahu et al., 2019). However, Dox-induced cardiomyocyte apoptosis is associated with NF-κB activation (Wang et al., 2002), and myocardial apoptosis is the predominant mode of Dox-induced cardiomyocyte death in in vitro and *in vivo* models (Christidi and Brunham, 2021).

### 3.5 Autophagy

Autophagy is an important mode of energy catabolism in the organism (Gatica et al., 2015), whose main function is to recycle damaged or unwanted cellular components, thereby maintaining cell viability, and is often considered a protective mechanism (Koleini and Kardami, 2017). Autophagy is involved in maintaining intracellular homeostasis in most types of cardiovascular cells, especially cardiomyocytes (Taneike et al., 2010; Gottlieb and Mentzer, 2013; Xiao et al., 2019; Luan et al., 2021; Elshazly et al., 2022). Several studies have shown that under normal conditions, decreased levels of autophagy are an adaptive response of the body to the heart to prevent cardiomyocyte death (Mei et al., 2015). However, cardiomyocyte autophagy dysfunction is closely associated with the development of arrhythmias (Luan et al., 2021). Autophagy, as a regulatory mechanism, is highly conserved throughout the life cycle of yeast and humans (Bestion et al., 2023), and plays a large role in tumorigenesis (Amaravadi et al., 2016; Udristioiu and Nica-Badea, 2019; Zhang et al., 2023). Anthracycline-induced myocardial damage is closely related to autophagy (Koleini and Kardami, 2017). Indeed, Dox promotes cardiac autophagy and contributes to the pathogenesis of the resulting cardiotoxicity (Wang et al., 2014), and autophagy dysfunction is an important cause of excessive cardiomyocyte death (Bartlett et al., 2017). It has been shown that dysregulated (or excessive) autophagy can be inhibited to achieve a reduction in DOX toxicity (Yin et al., 2018; Lv et al., 2020). The phosphatidylinositol 3-kinase/Akt/mammalian target of the rapamycin (PI3K/Akt/mTOR) pathway is thought to be a central regulatory element in the autophagy process (Sun et al., 2013). Dox regulate upstream regulatory aspects of autophagy, such as mTOR, AMPK, etc., whereas PI3K is also activated in Dox cardiomyotoxic rats (Bartlett et al., 2017).

### 3.6 Inflammation

Continued and uncontrolled inflammatory response can result in cardiac failure, arrhythmias, and even sudden death (Liu et al., 2016; Hiram et al., 2021). 2019 Novel coronavirus (COVID-19) is an important systemic systemic inflammatory disease that is closely associated with the onset and development of several cardiac arrhythmias (Gawałko et al., 2020; Coromilas et al., 2021). Several studies have confirmed that inflammation is strongly linked to AF, and inflammatory signaling in atrial cardiomyocytes is activated in both animal experimental models and in patients with AF (Elahi et al., 2008; Patel et al., 2010; Pastori et al., 2018; Dobrev et al., 2023). NF-κB has been suggested to be a key transcriptional regulator of pro-inflammatory factors (Tam et al., 2012). The accumulation of large amounts of ROS further triggers the NF-κB pathway to release inflammatory factors such as TNF-α and IL-6 (Rahman, 2002), triggering a series of inflammatory responses (Abd El-Aziz et al., 2012). Inflammatory changes are common in AF triggered by tumor therapy, and it has been shown that inflammation may be a hidden mechanism of Dox-induced cardiotoxicity (Thandavarayan et al., 2015; Monkkonen and Debnath, 2018). Dox significantly activates the NF-κB signaling pathway, which induces myocardial inflammation in mice [9]. During the course of chemotherapy with gemcitabine *versus* vincristine, the presence of atrial flutter and AF is more common, which is associated with the activation of the NF-κB pathway (Qanungo et al., 2014). Growing evidence suggests that melphalan has the potential to trigger the NF-κB pathway and increase pro-inflammatory cytokines in myocardial tissue, further triggering the development of arrhythmias (Qing et al., 2006; Baumann et al., 2008; Salminen et al., 2012; Amin et al., 2020; Lan et al., 2020; Rawat et al., 2021). Inflammation is a key factor in cisplatin-induced cardiotoxicity, and increasing evidence supports that cisplatin enhances the release of inflammatory cytokines and chemokines, and that inflammation-associated genes are mainly regulated by the transcription factor NF-κB. NF-κB activation in cisplatin-induced cardiac injury leads to the expression of tumor necrosis factor α in cardiomyocytes, causing cardiac remodeling (Chowdhury et al., 2016; Manohar and Leung, 2018). The signaling pathway of Toll-like Receptor 4 (TLR4) plays a central role in Dox-induced cardiac inflammation (Sumneang et al., 2023), which may be consistent with the mechanism of arrhythmia induced by ibrutinib.

## 4 Effects of Chinese herbal medicine and its Constituent compounds against arrhythmogenic injury induced by chemotherapeutic agents

Malignant tumors and cardiovascular diseases are two of the most common major diseases in the world today, and cardiovascular toxicity brought about by chemotherapeutic drug treatment has become an important threat to the life and safety of cancer patients. In particular, secondary CDIA caused by chemotherapeutic agents are very common. In recent years, it has been found that traditional Chinese medicine has the ability to antagonize the myocardial injury caused by chemotherapeutic drugs without affecting the efficacy of antitumor drugs. However, the mechanism of Chinese herbal medicines against chemotherapy drug-induced myocardial injury and potential intervention with the secondary arrhythmia is still not fully elucidated. In this regard, we have reviewed the research on chemotherapy-induced arrhythmogenic cardiotoxicity in Chinese herbal medicines and their components at home and abroad to provide a theoretical basis for the clinical application of Chinese herbal medicines to prevent and treat chemotherapy-induced myocardial toxicity (Figure 2; Table 2).

**TABLE 2:** Effects and mechanisms of TCM on chemotherapy-related myocardial injury and resultant arrhythmia.

Agents	TCM	Mechanism of action		Function			Type of study	Other cardiotoxicity endpoints	References
Dox	CUR	Bcl-2**↑**, caspase-8**↓**, caspase-9**↓**		Apoptosis			*in vitro*, *in vivo*		Mohajeri and Sahebkar (2018)
Dox		ROS**↓**		Oxidative stress			*in vitro*	Heart failure, cardiomyopathy	Jain and Rani (2018), Sheu et al. (2015)
Dox		COX-2**↓**, iNOS**↓**, NF-κB**↓**		Inflammation			*in vivo*		Farhood et al. (2019)
Dox	DSS	Keap1**↓**, Nrf2**↑**,NQO1**↑**		Apoptosis; oxidative stress; inflammation			*in vitro*, *in vivo*	heart failure, Myocardial hypertrophy	Qi et al. (2022), Tang et al. (2011), Yin et al. (2013)
Dox		TNF-α**↓**, IL-6**↓**; Bax**↓**, Bcl-2**↑**		Inflammation; apoptosis			*in vitro*, *in vivo*		Qi et al. (2022)
Dox	CAR	Caspase-3**↓**, NF-κB**↓**		Apoptosis; inflammation			*in vitro*, *in vivo*	myocardiainjuries	Qin et al. (2012), Abu Gazia and El-Magd (2018)
Dox		Nrf2↑		Inflammation			*invitro*, *in vivo*	myocardiopathy	Li et al. (2016), Qi et al. (2020)
Dox	Ginsenosides	Rb1		Caspase-3**↓**,caspase-8**↓**		Apoptosis	*in vitro*	myocardiainjuries	Zhang et al. (2017)
Dox		Rg3		Akt↑, Nrf2-ARE↑		Oxidative stress, apoptosis	*in vitro*, *in vivo*	Left ventricular systolic dysfunction	Wang et al. (2015), Li et al. (2017)
Dox		Rg1		JNK1**↓**		Endoplasmic reticulum stress	*in vivo*	Cardiomyopathy, Left ventricular systolic dysfunction	Xu et al. (2018)
CP	QUE	Nrf2↑, HO-1↑		Oxidative stress			*in vitro*, *in vivo*	congestive heart failure, cardiomyopathy	Wang et al. (2022); Sharma et al. (2020)
CyC		ROS**↓**		Oxidative stress			*in vitro*, *in vivo*		Zhang et al. (2022)
AC		ERK1/2↑		Oxidative stress			*in vitro*, *in vivo*		Zhang et al. (2022)
Dox		AMPK↑		Myocardial energy metabolism			*in vivo*		Zakaria et al. (2018)
Dox		p53**↓**		Apoptosis			*in vitro*, *in vivo*	cardiac dysfunction	Dong et al. (2014)
Dox		TNF-α**↓**		Inflammation			*in vivo*	myocardial lesions	Matouk et al. (2013)
Dox	QSHWC	PI3K/Akt↑,MAPK↓,FOXO↓		Apoptosis			*in vitro*, *in vivo*	left ventricular dysfunction	Wang et al. (2021)
Dox		p53**↓**		Apoptosis			*in vitro*, *in vivo*	left ventricular dysfunction	Xiao et al. (2012)
Dox		Bcl-2**↑**, Bax**↓**		Apoptosis			*in vivo*	congestive heart failure	Sahu et al. (2016)
THP		PI3K/Akt/mTOR**↑**		Autophagy			*in vivo*	myocardial injury	Wang et al. (2020)
Dox		PI3K/Akt↑, MAPK**↓**		Oxidative stress, autophagy			*in vitro*, *in vivo*	left ventricular dysfunction	Wang et al. (2021)
Dox	TMYXP	Nrf2/HO-1**↑**,p38-MAPK**↓**		Apoptosis			*in vivo*		Cui et al. (2018)
Dox		MAPK**↓**, p53**↓**		Apoptosis			*in vitro*, *in vivo*		Shu et al. (2023)
Dox	CDDP	TNFα**↓**,TGFβ1**↓**, αSMA**↓**		Inflammation			*in vivo*	Heart failure	Feng et al. (2021)
Dox		p-AMPK**↑**,Nrf2**↑**,ROS**↓**,FFA**↓**		Oxidative stress			*in vivo*	Heart failure	Feng et al. (2021)
Dox		Bax**↓**		Apoptosis			*in vivo*	Heart failure	Feng et al. (2021)
Dox	SQFZI	SIRT3**↑**,ERS**↓**		Endoplasmic reticulum stress			*in vitro*, *in vivo*	cardiomyopathy, cardiac dysfunction	Zhang et al. (2022)
Dox		microRNA**↑**,MAPK**↓**		Apoptosis			*in vitro*, *in vivo*		Rao et al. (2023)
Dox	SMI	AMPK**↑**, PI3K/Akt/GSK-3β**↑**		Oxidative stress, apoptosis			*in vitro*, *in vivo*	cardiomyocyte death	Li et al. (2020)
Dox		PI3K/Akt**↑**		Apoptosis			*in vitro*, *in vivo*		Li et al. (2020), Zhang et al. (2021)
Dox		JNK-mTOR**↓**		Apoptosis, autophagy			*in vitro*		Wu et al. (2021)
Dox	SgMI	Akt**↑**, ERK1/2**↓**		Oxidative stress			*in vivo*	heart dysfunction	Zhu et al. (2019)
Dox		caspase-12**↓**		Endoplasmic reticulum stress, apoptosis			*in vivo*	heart dysfunction	Chen et al. (2015)
Dox	XMLI	HO-1**↑**		Oxidative stress			*in vitro*		Jiang et al. (2021)
Dox		PI3K/Akt**↑**, ERK1/2**↓**, P38-MAPK↓, PKB/Akt**↑**,PI3K**↑**, Bcl-2**↑**		Autophagy			*in vivo*	Heart failure	Li et al. (2016)

DOX, doxorubicin; CP, cisplatin; CyC, cyclophosphamide; AC, the combination of doxorubicin and cyclophosphamide; THP, pirarubicin; CUR, curcumin; DSS, danshensu; CAR, cardamonin; QUE, quercetin; QSHWC, qishen huanwu capsule; TMYXP, tongmai yangxin pill; CDDP, compound danshen dropping pill; SQFZI, shenqi fuzheng injection; SMI, ShenMai injection; SgMI, shengmai injection; XMLI, xinmailong injection.

### 4.1 Chinese medicinal compounds

#### 4.1.1 Curcumin

Curcumin (CUR) is a naturally occurring polyphenol derived from the turmeric plant (Yu et al., 2019), that is insoluble in water (Farhood et al., 2019; Najafi et al., 2020). This natural polyphenol is the key active substance in turmeric (Wang et al., 2015), which is a natural antioxidant (Bahadır et al., 2018). CUR exhibits a variety of biological and pharmacological properties, and the protection of cardiomyocytes by non-toxic doses of CUR is mainly achieved by enhancing its antioxidant function, modulating cell death, and exerting anti-inflammatory effects (Jain and Rani, 2018; Mohajeri and Sahebkar, 2018; Armandeh et al., 2022; Zhang and Wu, 2022). CUR is a well-known anticancer drug that regulates cell proliferation and apoptosis at multiple levels (Mohajeri and Sahebkar, 2018). CUR reduces the side effects of chemotherapy and improves the sensitivity of chemotherapeutic agents (Zhang and Wu, 2022). Pretreatment with CUR can significantly alleviate Dox-induced cardiomyocyte death by increasing Bcl-2 and decreasing caspase-8 and caspase-9 levels (Mohajeri and Sahebkar, 2018). The Bax/Bcl-2 ratio was significantly decreased after 2 h of CUR pretreatment; furthermore, CUR inhibited Dox-mediated ROS generation (Sheu et al., 2015; Jain and Rani, 2018). CUR also acts on a variety of inflammatory mediators, such as cyclo-oxygenase-2 (COX-2), inducible nitric oxide synthase (iNOS), and NF-κB, to reduce the secretion of inflammatory factors, decrease chronic free radical production and attenuates tissue toxicity (Farhood et al., 2019). However, contradictory findings were reported by Hosseinzadeh et al. (2011), with the *in vitro* study results showing that pretreatment with CUR at nontoxic concentrations (5–15 µM) for 1 h significantly potentiated DOX-induced apoptosis in rat H9c2 cardiac muscle cells through downregulation of Bcl-2 (an increase in Bax/Ccl-2 ratio), upregulation of caspase-8 and caspase-9, and an increase in ROS generation by Dox. Potentiation of Dox-induced death of H9c2 cells was also confirmed by Jain and Rani (2018) when treating CUR with DOX concurrently, but not after pretreatment of CUR. Therefore, the clinic use of CUR, especially the dosing regimen, to mitigate Dox-induced cardiotoxicity in cancer patients needs to be re-evaluated.

#### 4.1.2 Danshensu

Danshen (Salvia miltiorrhiza Bge) is the root and rhizome of the herb Danshen in the family Labiatae, which is commonly used clinically against heart disease (Wang et al., 2017). Danshensu (DSS) is a water-soluble substance in Salvia miltiorrhiza, which mainly consists of catechol and lactic acid (Zhang et al., 2019). DSS has various pharmacological activities, such as antioxidant, anti-apoptotic, anti-myocardial ischemia, and attenuation of myocardial hypertrophy (Tang et al., 2011; Yin et al., 2013; Bao et al., 2018). Nrf2 deficiency aggravates Dox-induced myocardial toxicity and cardiac dysfunction (Bellezza et al., 2018; Wallace et al., 2020). Network pharmacological studies suggest that Keap1-Nrf2/NQO1 is a key factor contributing to Dox-induced cardiotoxicity. In animal experiments, DSS was able to effectively counteract Dox-induced cardiotoxicity by regulating the expression of Keap1-Nrf2/NQO1, exerting antioxidant, anti-inflammatory, and anti-apoptotic therapeutic effects, and thus attenuating Dox-induced cardiac injury (Yin et al., 2013; Qi et al., 2022). Literature reports that TNF-α, IL-6, *etc.*, can induce inflammatory responses, and DSS alleviates Dox-induced myocardial injury by decreasing the expression of TNF-α, IL-6, etc. In addition, DSS effectively alleviated Dox-induced cardiomyocyte apoptosis, mainly by preventing the upregulation of Bax and the downregulation of Bcl-2. (Qi et al., 2022).

#### 4.1.3 Cardamonin

Cardamonin (CAR) is a flavonoid compound isolated from traditional Chinese medicine and is widely found in a variety of herbs such as Myristica fragrans, Ginkgo biloba, and hickory, etc. This flavonoid compound has various pharmacological activities such as anti-inflammatory (Lee et al., 2006; Lee et al., 2012; Li et al., 2015; Wang et al., 2018), anti-tumor (Qin et al., 2012), anti-OS(Bajgai et al., 2011; Peng et al., 2017; Tan et al., 2021), etc. In different cells, CAR has different regulatory effects. In cardiomyocytes, CAR has been found to have antioxidant effects, which can inhibit the generation of ROS, induce apoptosis, and prevent cells from oxidative stress and inflammatory damage by up-regulating signaling pathways such as Nrf2 and NF-κB, thus protecting cardiomyocytes (Qi et al., 2020; Tan et al., 2021). In tumor cells, CAR can promote oxidative stress. CAR can inhibit the proliferation and migration of tumor cells by inhibiting the expression of NF-κB, subsequently enhancing the oxidative phosphorylation level of mitochondria, increasing the accumulation of ROS in tumor cells, inducing cell cycle arrest and apoptosis (Jin et al., 2019). CAR attenuates Dox-induced cardiomyocyte cytotoxicity by inhibiting apoptosis through downregulation of the Caspase-3, NF-κB pathway, and suppressing the inflammatory response (Qin et al., 2012; Abu Gazia and El-Magd, 2018). In the study of the Dox-triggered cardiotoxicity animal model, we found that by activating the Nrf2 signaling mechanism, CAR could effectively inhibit the OS response within the mouse myocardium, slow down the death of apoptotic cells, and inhibit the inflammatory response, which ultimately contributed to the reduction of myocardial injury (Li et al., 2016; Qi et al., 2020).

#### 4.1.4 Ginsenosides

Ginsenosides are the most crucial active substances in ginseng, which have antioxidant, apoptosis inhibiting, autophagy regulating, and other cardiotoxicity-reducing effects (Hou et al., 2022; Lv et al., 2022). It has been found that Dox can cause apoptosis and autophagy in H9c2 cardiomyocytes, and ginsenoside Rb1 can attenuate Dox-induced myocardial injury by a mechanism that may be realized by inhibiting Dox-induced autophagy (Li et al., 2017; Zhang et al., 2017; Zhu et al., 2017). Meanwhile, Rb1 not only reduces the activities of caspase-3 and caspase-8 but also interrupts the apoptotic process of H9C2 cells (Zhu et al., 2017). Ginsenoside Rg3 (Rg3) is an anticancer active component of ginseng. According to studies, it possesses antioxidant, anti-apoptotic, and cardioprotective effects, and its mechanism of action is related to Akt activation and exerts antioxidant effects and inhibits apoptosis through activation of the Nrf2-ARE pathway (Wang et al., 2015; Li et al., 2017). Rg1 attenuates DOX-induced endoplasmic reticulum stress and autophagy in the hearts of mice by inhibiting the JNK1 signaling pathway (Xu et al., 2018).

#### 4.1.5 Quercetin

Quercetin (QUE) is a polyhydroxyflavonoid widely found in plants and animals, and an increasing number of studies have confirmed its ability to effectively inhibit mitochondrial OS, suppress myocardial fibrosis, inhibit cardiomyocyte apoptosis, and improve myocardial remodeling (Zhou et al., 2022). QUE attenuates cisplatin and Dox-induced myocardial injury by activating the expression of Nrf 2, HO-1 (Sharma et al., 2020; Wang et al., 2022). QUE exhibits excellent antioxidant properties, which can reduce cyclophosphamide (CyC)-induced cardiotoxicity by inhibiting ROS accumulation in cardiomyocytes and further attenuates cardiotoxicity caused by the combination of doxorubicin and cyclophosphamide (AC) by activating the ERK1/2 pathway (Zhang et al., 2022). QUE can upregulate the expression of AMPKα2, PPARα, and PGC-1α by regulating the AMPK pathway, improving myocardial energy metabolism and preventing Dox-induced myocardial injury in rats (Zakaria et al., 2018). Studies have shown that QUE reduces the effect of Dox on p53 expression, which may, to some extent, account for the attenuation of Dox-induced apoptosis by QUE (Dong et al., 2014). QUE reduces Dox-induced TNF-α, leading to a protective effect on rat cardiomyocytes (Matouk et al., 2013). QUE has bidirectional pharmacological effects on cardiomyocytes, as evidenced by the fact that low-dose QUE exerts cardioprotective effects by potentiating the activity of ERK1/2 and then modulating its target genes (Angeloni et al., 2007), whereas high-dose QUE exerts cardiotoxic effects by inhibiting the activity of ERK1/2 and then regulating its target genes to exert cardiotoxic effects (Daubney et al., 2015).

### 4.2 Chinese medicine Combination Formula

#### 4.2.1 Qishen Huanwu Capsule

Qishen Huanwu Capsule (QSHWC) is a compound preparation mainly composed of Huangqi (Astragalus), Taizishen (Pseudostellaria heterophylla), Chuanxiong (Ligusticum chuanxiong), Danggui (Angelica sinensis), Chishao (Paeonia lactiflora), Taoren (Persica), Honghua (Carthamus tinctorius), Niuxi (Achyranthes bidentata), Banxia (Pinellia ternata), and Maidong (Ophiopogon japonicus). Its main components, quercetin (Chen et al., 2019), kaempferol (Xiao et al., 2012), and isorhamnetin (Sun et al., 2013) have significant anti-anthracycline cardiotoxicity effects. Kaempferol downregulates the expression of p53 and binds to the promoter of pro-apoptotic genes, thereby attenuating Dox-induced OS, apoptosis, and mitochondrial damage (Xiao et al., 2012). Baicalein, the main active substance in QSHWC, counteracts Dox-induced myocardial toxicity by enhancing the expression of Bcl-2, decreasing the expression of Bax, decreasing the ratio of Bax to Bcl-2, and inhibiting apoptosis in cardiomyocytes (Sahu et al., 2016). QSHWC effectively alleviated myocardial toxicity caused by pirarubicin (THP), which was mainly closely related to the initiation of the PI3K/Akt/mTOR pathway and the reduction of THP-induced excessive autophagy in cardiomyocytes (Wang et al., 2020). Qishen Huanwu Capsule regulates PI3K/Akt, MAPK and FOXO signaling pathways by regulating targets such as Akt1, MAPK1, MAPK8 and thereby inhibits oxidative stress and regulates apoptosis and autophagy to reduce the cardiotoxicity of anthracyclines. (Wang et al., 2021).

#### 4.2.2 Tongmai Yangxin Pill

Tongmai Yangxin Pill (TMYXP) is a traditional Chinese medicine (TCM) that primarily contains ingredients such as Dihuang (Rehmannia glutinosa), Jixueteng (Millettia speciosa), Maidong (Ophiopogon japonicus), Gancao (Glycyrrhiza uralensis), Zhi Heshouwu (Polygonum multiflorum), Ejiao (Donkey-hide gelatin), Wuweizi (Schisandra chinensis), Dangshen (Codonopsis pilosula), Cuguojia (Testudinis Carapax Et Plastrum prepared with vinegar), Dazao (Ziziphus jujuba), and Guizhi (Cinnamomum cassia). After preliminary activity screening through various pharmacological studies, 80 compounds were identified from TMYX pills, including flavonoids, coumarins, iridoid glycosides, saponins and lignans, which had significant anti-inflammatory and antioxidant activities (Fan et al., 2016). OS response is an important biological process in which TMYXP plays a role, and its mechanism of action may be related to the Nrf2 and MAPK pathways. It was found that TMYXP could enhance anti-OS ability and reduce apoptosis by regulating signaling pathways such as Nrf2/HO-1 and p38-MAPK(Cui et al., 2018). Network pharmacology and metabolomics studies suggest that TMYXP can attenuate Dox-induced myocardial injury by regulating the upstream protein signaling pathway of insulin, MAPK, p53, and other signaling pathways, as well as regulating energy metabolism (Shu et al., 2023).

#### 4.2.3 Compound Danshen Dropping Pill

Compound Danshen Dropping Pill (CDDP) is a well-known formula commonly used clinically against cardiovascular diseases (Feng et al., 2021). The three herbal ingredients include Danshen (Salvia miltiorrhiza), Sanqi (Panax notoginseng), and Bingpian (Borneol) (Guo et al., 2016; Hu et al., 2022), and it was found that its main active ingredients are danshenolic acids (e.g., danshenolic, etc.), saponins (e.g., ginsenoside Re), flavonoids (e.g., Tan IIA), and icariin, etc., CDDP treatment of rabbits with surgically induced acute myocardial infarction significantly inhibits cardiac apoptosis, reduces oxidative stress and inflammation, and improves cardiac function (Jun et al., 2014). CDDP can effectively reduce the level of Dox-induced serum TNFα and its expression in the heart (Feng et al., 2021). Experiments have demonstrated that CDDP can effectively reverse the inhibitory effect of Dox on p-AMPK (the active form of AMPK) and downregulate the expression of Nrf2, which in turn reduces ROS and FFA production, counteracts Dox-induced OS, and attenuates myocardial injury. Expression of transforming growth factor β1 (TGFβ1) and αsmooth muscle actin (αSMA), key mediators of fibrosis, was significantly increased in Dox-treated mice, and CDDP could significantly ameliorate Dox-induced myocardial fibrosis and inflammation and reduce the risk of cardiovascular injury by suppressing the expression of pro-fibrotic and pro-inflammatory molecules (Feng et al., 2021). CDDP was able to counteract Dox-induced cardiomyocyte apoptosis *in vivo* by modulating the expression of Bax protein (Feng et al., 2021).

### 4.3 Chinese medicine injection

#### 4.3.1 Shenqifuzheng injection

The main components of Shenqi Fuzheng Injection (SQFZI) are Radix et Rhizoma Ginseng and Radix Astragali (Chen et al., 2019), which have the efficacy of benefiting qi and strengthening qi. SQFZI may play a protective role by increasing myocardial energy metabolism, inhibiting cell adhesion, suppressing inflammatory responses, reducing cardiomyocyte apoptosis, and preventing myocardial remodeling (Liao et al., 2018). Among the anti-cardiotoxic components of SQFZI, several members have been clarified in published studies to date. For example, ononin (a natural isoflavone glycoside), ononin has a significant antimyocardial injury effect, primarily by attenuating the effects of Dox treatment on cardiomyocyte apoptosis through activation of SIRT3 and inhibition of ER stress (Zhang et al., 2022). The role of SQFZI in combating cardiac injury is also related to microRNAs, MAPK and other signaling pathways (Rao et al., 2023). microRNAs (miRNAs) play a key regulatory role in cell proliferation, cell death, apoptosis and cell differentiation, and their dysregulation is closely related to cardiotoxicity (Pellegrini et al., 2020). It has been shown that blocking the MAPKDEBE signaling pathway modulates apoptosis, thereby attenuating myocardial toxicity (Zhang et al., 2021).

#### 4.3.2 ShenMai injection

ShenMai injection (SMI) consists of Renshen (Ginseng Radix) and Maidong (Ophiopogonis Radix), which is derived from Shengmaisan in the Thousand Golden Essentials (Li et al., 2020). AMPK is central to Dox-induced cardiotoxicity events (Timm and Tyler, 2020). *In vitro*, SMI rescued Dox-injured H9c2 cardiomyocytes from apoptosis, mitochondrial ROS overproduction, and mitochondrial membrane potential depletion. In addition, SMI prevents Dox-induced cardiotoxicity by inhibiting mitochondrial oxidative stress and fragmentation through activation of AMPK and PI3K/Akt/GSK-3β signaling pathways (Li et al., 2020). SMI increased the viability of Dox-injured H9c2 cardiomyocytes and prevented apoptosis, and its protective effect against cardiotoxicity may be related to the activation of the PI3K/Akt pathway (Li et al., 2020; Zhang et al., 2021). SMI regulates cardiomyocyte apoptosis and autophagy by controlling the JNK (a unique autophagy-activating signal)-mTOR signaling pathway and blocking the Dox-induced apoptotic pathway and autophagy formation (Wu et al., 2021).

#### 4.3.3 ShengMai injection

Shengmai injection (SgMI), a traditional Chinese medicine extracted from shanghai san including Renshen (Ginseng Radix), Maidong (Ophiopogonis Radix), and Wuweizi (Schisandra Chinensis Fructus) (Wang et al., 2020). It prevents cardiotoxicity of chemotherapy drugs by enhancing myocardial contractile function, reducing afterload and arrhythmia (Mao and Xu, 2007; Zhang and Zhang, 2007). SgMI alleviates myocardial damage and cardiac dysfunction in Dox-treated patients (Yang, 2008). SgMI alleviates cardiac damage and cardiac dysfunction by regulating TLR4, NF-κB, and other protein expression to reduce downstream inflammatory factor expression and attenuate cardiac damage (Qing-min et al., 2018). Studies have shown that SgMI attenuates OS-induced cardiomyocyte injury through Akt and ERK1/2 pathways (Zhu et al., 2019). SgMI can help ameliorate Dox-induced cardiomyocyte injury in rats by alleviating myocardial ER stress and ER stress specific apoptosis through inhibition of caspase-12-dependent pathways (Chen et al., 2015).

#### 4.3.4 Xinmailong Injection

Xinmailong Injection (XMLI) is a bioactive compound extracted from the cockroach (a species of cockroach), which has been found to have favorable anti-cardiovascular damaging effects (Li et al., 2016). XMLI protects against Dox-induced myocardial injury by a mechanism that is mediated by HO-1 regulation of lysosomal function and improvement of autophagic flux, and reduces OS(Jiang et al., 2021). Findings have demonstrated that XML also inhibits cellular autophagy and attenuates Dox-induced cardiac damage through signaling pathways such as activation of PI3K/Akt, inhibition of ERK1/2, P38 MAPK, etc .,(Li et al., 2016). Beclin 1 has been identified as a Bcl-2-interacting protein and is significant for autophagy. XML attenuates the accumulation of Beclin1 with Atg7, increases protein kinase B (PKB)/Akt, PI3K, and Bcl-2 expression, inhibiting autophagy and ameliorating myocardial injury (Li et al., 2016).

## 5 Conclusion

In summary, based on the current literature review, the main types of secondary CDIA are atrial fibrillation (paroxysmal, persistent), ventricular arrhythmias (premature ventricular contraction, ventricular tachycardia, ventricular fibrillation) and sinus bradycardia (atrioventricular block). The mechanism of arrhythmia induced by chemotherapy drugs is very complex, mainly including oxidative stress, myocardial energy metabolism, endoplasmic reticulum stress, apoptosis, autophagy and inflammation. Chemotherapy drugs have the potential to induce cardiac toxicity, leading to conditions such as heart failure, cardiomyopathy, and myocardial injury through the above various pathways. Substances generated from myocardial damage or cell death caused by chemotherapy-related cytotoxicity serve as substrates for arrhythmias, contributing to the initiation and progression of arrhythmias. Chinese herbal medicine has been identified as having the capability to mitigate the adverse effects of chemotherapy drugs through diverse mechanisms. Specifically, certain Chinese herbal medicines, including CUR, DSS, QUE, QSHWC, CDDP, SMI, SgMI, and XMLI, demonstrate anti-oxidative stress properties. Additionally, compounds such as CUR, DSS, CAR, QUE, and CDDP exhibit anti-inflammatory effects, while CUR, DSS, CAR, QUE, QSHWC, TMYXP, CDDP, SQFZI, SMI, and SgMI are associated with anti-apoptotic properties. Some herbal medicines, including QSHWC, SMI, and XMLI, play a role in regulating autophagy, and others, such as SQFZI, and SgMI, contribute to the regulation of endoplasmic reticulum stress. Moreover, QUE has been recognized for its ability to enhance myocardial energy metabolism. Collectively, these herbal medicines function through diverse pathways to attenuate the arrhythmogenic toxicity induced by chemotherapy drugs. These pathways encompass the regulation of Keap1-Nrf2/NQO1 expression, prevention of the upregulation of Bax and downregulation of BCL-2 in mitochondrial apoptosis procedures. They also involve the promotion of the expression of AMPKα2, PPARα, and PGC-1α, activation of the PI3K/Akt signaling pathway, inhibition of the ERK1/2 and P38MAPK signaling pathways, and enhancement of Nrf2 expression. More importantly, Chinese medicine can play the role of anti-arrhythmia caused by chemotherapy drugs without affecting the anti-cancer effect of chemotherapy drugs. The limitations of this article are: up to now, there are few literature on the treatment of specific types of CDIA by certain Chinese medicine compounds or injections. This review first summarizes common types of CDIA, such as atrial fibrillation, ventricular arrhythmia, and sinus bradycardia. The potential intervention of CDIA with TCMs may not be limited to the above mentioned three arrhythmia. Prevention and/or treatment of specific type of CDIA with TCMs will be reviewed in the future once the dataset from clinical studies and basic research becomes available.
